# Evaluating the comparative efficiency of medical centers in Taiwan: a dynamic data envelopment analysis application

**DOI:** 10.1186/s12913-022-07869-8

**Published:** 2022-04-02

**Authors:** Cheng-Ming Chiu, Ming-Shu Chen, Chung-Shun Lin, Wei-Yu Lin, Hui-Chu Lang

**Affiliations:** 1 Department of Rehabilitation, Far Eastern Memorial Hospital, No.21, Sec. 2, Nanya S. Rd., Banciao Dist., New Taipei City, 220 Taiwan; 2 Department of Accounting, Far Eastern Memorial Hospital, No.21, Sec. 2, Nanya S. Rd., Banciao Dist., New Taipei City, 220 Taiwan; 3 Department of Healthcare Administration, College of Healthcare & Management, Asia Eastern University of Science and Technology, No.58, Sec. 2, Sichuan Rd., Banciao Dist., New Taipei City, 220 Taiwan; 4 Institute of Hospital and Health Care Administration, National Yang Ming Chiao Tung University, No. 155, Sec.2, Li-Nong St., Taipei, 112 Taiwan

**Keywords:** Data envelopment analysis, Dynamic efficiency, Projections, Beta regression, Hospital performance

## Abstract

**Background:**

People in Taiwan enjoy comprehensive National Health Insurance coverage. However, under the global budget constraint, hospitals encounter enormous challenges. This study was designed to examine Taiwan medical centers' efficiency and factors that influence it.

**Methods:**

We obtained data from open sources of government routine publications and hospitals disclosed by law to the National Health Insurance Administration, Ministry of Health and Welfare, Taiwan. The dynamic data envelopment analysis *(*DDEA) model was adopted to estimate all medical centers' efficiencies during 2015–2018. Beta regression models were used to model the efficiency level obtained from the DDEA model. We applied an input-oriented approach under both the constant returns-to-scale (CRS) and variable returns-to-scale (VRS) assumptions to estimate efficiency.

**Results:**

The findings indicated that 68.4% (13 of 19) of medical centers were inefficient according to scale efficiency. The mean efficiency scores of all medical centers during 2015–2018 under the CRS, VRS, and Scale were 0.85, 0.930, and 0.95,respectively. Regression results showed that an increase in the population less than 14 years of age, assets, nurse-patient ratio and bed occupancy rate could increase medical centers' efficiency. The rate of emergency return within 3-day and patient self-pay revenues were associated significantly with reduced hospital efficiency (*p* < 0.05). The result also showed that the foundation owns medical center has the highest efficiency than other ownership hospitals.

**Conclusions:**

The study results provide information for hospital managers to consider ways they could adjust available resources to achieve high efficiency.

**Supplementary Information:**

The online version contains supplementary material available at 10.1186/s12913-022-07869-8.

## Background

In 1995, Taiwan launched National Health Insurance (NHI) that covers 99% of Taiwan's 23 million people with a single-payer universal healthcare scheme that ensures that every resident has access to quality, affordable medical care. The comprehensive coverage includes inpatient, outpatient, dental, and home nursing care, prescription drugs, and traditional Chinese medicine. In addition, 92% of clinics and hospitals are contracted to NHI, and patients have a free choice of doctors or hospitals. Hence, patients can go directly to a medical center with a very low extra copayment [1]. Initially, the Bureau of National Health Insurance (BNHI) reimbursed healthcare providers on a fee-for-service basis and then implemented a global budget system gradually to each of the major healthcare sectors covered. In addition, the Diagnosis-Related Group payments system in Taiwan (Tw-DRG) was introduced in 2009 to control costs and enhance medical efficiency under the universal coverage, single-payment insurance system: National Health Insurance (NHI).

According to the Taiwan Statistics of Medical Care Institution's Status & Hospital Utilization 2018, the number of beds medical centers owned accounts for 24% of the total number of beds, 44% of doctors, and 34% of non-physician medical staff. These resources are used to cover 27% of the entire hospitalization days in Taiwan (https://www.mohw.gov.tw/lp-4932-2.html). However, different medical centers were established and developed from diverse backgrounds, and include public, private, and foundation hospitals. For example, among the foundation hospitals, the business philosophies differ because of the ownership background, and all may affect the hospitals' performance.

Increasing demands for greater accountability require managers to give more attention to hospital performance. Charnes and Banker proposed Data Envelopment Analysis (DEA) initially and this model is referred to commonly as a CCR model [2, 3]. It is a linear programming approach to measure and evaluate the relative efficiency of similar decision-making units (DMUs). DEA can manage multiple inputs and outputs simultaneously without any assumptions about the data distribution. The DMUs on the DEA frontier are those with maximum output levels for given input levels or minimum input levels for given output levels. DEA provides efficiency scores for individual units as their technical efficiency measure, with a score of one assigned to the frontier (efficient) units.

DEA has been a method suggested to evaluate the efficiency of decision-making units (DMUs) in different sectors, including the health sector [4, 5]. The classical DEA models—CCR and BCC—are the most popular models used to assess the efficiency of hospitals, other healthcare facilities, and healthcare systems worldwide. Based upon these models, hospital performance has been used popularly to compare an estimated efficient frontier comprising the best-performing hospitals [6, 7]. Leleu et al. adopted the DEA approach to investigate the efficiency of private hospitals in the United States and the factors that affect it and found that hospitals located in more competitive markets were more efficient than those located in less competitive markets [8]. Some European researchers have performed DEA to measure the efficiency of public healthcare systems and the healthcare industry in general [9, 10]. Jiang et al. employed the DEA model to evaluate hospitals' efficiency and effectiveness before and after healthcare reforms were implemented in China, and found that reform did not improve the efficiency of hospital operations in Chia to any great extent [11]. Nakata et al. adopted the standard DEA to calculate each surgeon's technical efficiency in Japan and then demonstrated the effect of surgeons' revenue as a proxy variable in technical efficiency results [12].

However, classical DEA models, which are referred to as static models, ignore time effects and the inefficiencies of an organization's internal processes [13]. Even when time has been considered, DEA models were used to evaluate the efficiency of each time period separately or each DMU was treated at a different time period as a separate unit [14, 15]. Thus, the traditional models ignore changes in efficiency over time and carry-over effects, and the connecting activities between terms are not accounted for explicitly [16]. Hence, performance analyses that address dynamic changes in efficiency over time are demanded in many applications. Färe et al. developed the DEA-based Malmquist productivity index with the CCR model [17, 18]. The DEA-based Malmquist productivity is a combined index that decomposes the productivity change in DMUs over time into catch-up and innovation (frontier-shift) effects. These models have inputs and outputs for each term, but they do not account explicitly for the effect of carry-over activities between two consecutive terms.

The Malmquist index is the most popular method to measure efficiency changes over time; however, this approach neglects carry-over activities between two consecutive terms, and focuses only on the local optimization in each period, even if these models can take into account the time change effect. In the real world, long-term planning and investment are always a subject of great concern to businesses; hence, a single-period optimization model cannot evaluate performance perfectly. To cope with the long-term issue, the dynamic DEA model incorporates carry-over activities into the model and allows us to measure period-specific efficiency based upon the long-term optimization during the entire period.

The theoretical concept of dynamic DEA, DDEA were introduced by Tone and Tsutsui [14, 19]. Compared to the classical DEA model, this model allows the transition elements between subsequent observations of activities and establishes the interdependence between periods. Thus, the DDEA model can quantify the dependence between periods attributable to dynamic factors using specific elements that include information, characteristics of organizational systems, their physical structure, etc. [20]. The advantage of DDEA is that it can use carry-over activities as constraints between periods in efficiency evaluation, which play a significant role in measuring efficiency during consecutive periods. Importantly, in this model, an output from one period is treated as an input for the following period.

Hung et al. employed the DDEA model to evaluate the performance of Taiwanese business groups [21]. Kawaguchi et al. estimated the dynamic changes in efficiency based upon current reforms’ policy effects in Japan's municipal hospitals [22]. Mariz et al. provided a detailed overview of DDEA models that included the characteristics of the DMUs, the analysis period, and input, output, and intermediate elements [15]. This overview explained the flexibility of DDEA applications in various sectors, such as industry, service companies (banks, hotels, hospitals, employment agencies), transport infrastructure (railways and harbors), etc. Thus, it is noticed that the numbers of DMUs and elements (inputs, outputs and intermediates) can vary according to studies. The above studies provided evidences that the DDEA analyses were appropriate for dynamic efficiency between periods.

The selection of inputs and outputs is crucial for efficiency estimation. In general, inputs should incorporate all necessary resources, and outputs need to describe the managerial objectives of the DMU. O'Neill et al. (2008) and Ozcan (2014) proposed similar guidelines to choose the inputs and outputs for the DEA analysis [23, 24]. They identified three major input categories as capital investment, labor, and other operating costs. On the output side, they introduced case-mix adjusted outpatient visits, admissions or discharges, and teaching for those hospitals engaged in medical education. Kohl (2019) reviewed 262 papers of DEA applications in healthcare that specifically focused on hospitals and found that the principal inputs are the number of beds, medical staff, and nurses while the principal outputs are outpatients, other/total cases, and inpatients [25]. Besides that, supplies have seen the highest growth in input category usage, including medical supply expenses, Pharmaceutical costs, and other operational expenses.

The National Health Insurance has implemented measures to reduce hospital outpatient visits in medical centers since 2018. The decreasing rate has been set by 2% each year until it reaches 10% in 5 years. Under Taiwan's single-payer, global budget health insurance system, hospitals encounter constraint finance in providing quality patient care. Accordingly, hospitals, particularly medical centers, must seek more efficient management actively. Although the DDEA model has theoretical advantages, few studies have applied it to measure healthcare institutions' performance. To improve the weakness of static analysis in previous studies, we adopted the DDEA method to evaluate all medical centers (DMUs) in Taiwan during 2015–2018. In this study, we also incorporated a general industry EBITDA measure (Earnings before Interest, Taxes, Depreciation and Amortization, EBITDA), which no previous research has used in analysis. The results will help hospital managers scrutinize their operation efficiency compared to their peers.

## Methods

### Data sources

We conducted a retrospective panel-data study including all nineteen medical centers in Taiwan. Data were obtained from open sources of government routine publications and hospitals disclosed by law to the National Health Insurance Administration. Other variables included financial data from hospitals' public financial statements and the quality indicators from the " national health insurance medical quality information disclosure network. " (https://www.nhi.gov.tw/AmountInfoWeb/TargetItem.aspx?rtype=2). The data were collected from 2015 to 2018. In addition, some county and city-level data were derived from the 2018 demographic data of the Global Information Network of the Household Registration, Ministry of the Interior (https://www.ris.gov.tw/app/en/3910) and Health Statistics on the current status of medical institutions and hospital medical service volume, the Ministry of Welfare (https://www.mohw.gov.tw/np-129-2.html).

### Data analysis

Descriptive and correlation analyses were applied to all data obtained from the sources, and input and output variables were chosen if both were highly correlated. The efficiency analysis consisted of two stages. In the first stage, the DDEA model was used to estimate the efficiency of medical centers during 2015–2018. In the second stage, regression-based models, specifically beta regression analysis, were used to model the efficiency level obtained from the first stage of factors that could influence the efficiency score. After correlation testing, three input variables were included: The number of doctors (I1), which represents the availability of human resources for healthcare services; the number of beds (I2) as a proxy indicator for capital input, and gross equipment expenditure (I3) as a proxy indicator for operating costs. Three output variables included: the total adjusted combined inpatient and outpatient revenues in NHI(100 million NTD) (O1); earnings before interest, taxes, depreciation, and amortization (EBITDA) index (O2), which represents medical income plus depreciation and amortization divided by net medical revenue;, and rate of emergency transfer to the inpatient stay over 48 h (O3). Further, surplus or deficit of appropriation of total revenue (C1) was adopted as a carry-over variable in the DDEA model to explore efficiency in the following years. We thought the combined revenues of inpatient and outpatients is a more precise index because it also adjusted the casemix. In addition, the total surplus/deficit reflects the overall past performance level that will carry over to the following term. Thus, Surplus/deficit could be treated as good carry-overs and bad carry-overs.

### Dynamic data envelopment analysis (DDEA) Model

Tone and Tsutsui (2010) and Tone and Tsutsui (2014) introduced the following concept of DDEA [14, 19]. DDEA deals with $n$ number of DMUs, in which each unit consists of $m$ number of inputs to produce $s$ number of outputs over time $T$. The efficiency analysis of DMUs is measured over the time period (i.e., year by year). In DDEA models, carry-overs play an important role in transferring the decisions between each consecutive time period. These carry-over variables are referred to as links that can be treated as fixed or mutable according to the objective function and were classified here into four categories: desirable (good), undesirable (bad), discretionary (fixed), and non-discretionary (free). An input-oriented model under both CRS and VRS assumptions was applied to estimate the efficiency score in this study. DEA solver v. 15.1 software was adopted for the analysis.

### Factors that affect hospital efficiency

DDEA efficiency scores can be used further to determine which factors affect medical centers' efficiency. The efficiency score lies between 0 and 1. If the value is close to one, it indicates that the medical center is efficient. Beta regression is appropriate for this type of efficiency distribution [26] Pirani, Zahiri, Engali, and Torabipour (2018). The equation for the efficiency prediction model is defined as follows:

$${E}_{j}={\beta }_{0}+{\beta }_{1}{x}_{j1}+{\beta }_{2}{x}_{j2}+\dots +{\beta }_{m}{x}_{jm}+{\varepsilon }_{j}$$

in which ${E}_{j}$ indicates the medical centers' efficiency scores, ${\varepsilon }_{j}\sim N\left(0,{\sigma }^{2}\right)$ indicates the error terms, ${\beta }_{1},{\beta }_{2},\dots {\beta }_{m}$ indicate coefficients of ${x}_{ji}$ independent variables, and ${\beta }_{0}$ denotes the constant. Efficiency scores based upon the DEA model were applied to the beta regression model as the dependent variable to determine influencing factors [27, 28]. The beta regression analysis was performed using the *betareg* package in R.

In addition to the variables analyzed earlier, we also examined broad internal and external factors, including year, population (E1), ratio of population over age 65 (E2), percentage of the population less than age 14 (E3), assets (E4), surplus or deficit of appropriation (E5), total revenues for inpatient and outpatient services form NHI (E6), rate of emergence return within 3-day (E7), same-day emergency return rate (E8), Case Mix Index- CMI (E9), nurse-patient ratio (E10), bed occupancy rate (E11), patient self-pay revenues (E12), and medical income margin (E13) as independent variables against the efficiency calculated by DDEA. Further, we conducted a subgroup analysis of the efficiencies in regions, ownership, and hospital gross income.

## Results

Table 1 presents the descriptive statistics of all variables selected from the 19 medical centers for each year as well as the overall period. The results indicated that the mean number of doctors, beds, and gross equipment expenditures increased from 2015 to 2018. The output variables of combined inpatient and outpatient revenues from NHI had the same increasing trend as inputs during the study period. On the other hand, the EBITDA values, and the rate of emergency transfer in-patient stay over 48 h fluctuated. The carry-over variable of total surplus/deficit showed an increased trend during the study period. The correlations among inputs, output, and carry-overs are presented in Table S1. All correlations were significant and ranged from 0.16 to 1 (*p* < 0.05). Thus, all inputs, outputs, and carry-overs were suitable for use in the DDEA model.

**Table 1:** Descriptive Statistics during 2015–2018

**Variables**^a^	**2015**		**2016**		**2017**		**2018**		**2015–2018**	
	**Average**	**SD**	**Average**	**SD**	**Average**	**SD**	**Average**	**SD**	**Average**	**SD**
I1	740	354	765	364	794	372	823	384	780	369
I2	1678	802	1684	797	1694	800	1698	795	1688	799
I3	3877.8	2208	4114.5	2285.1	4272.9	2371.2	4465.8	2469.3	4182.7	2333.4
C1	673.7	673	975.6	1226.8	1238.8	2046.9	1302.5	2130.7	1047.7	1519.4
O1	8922.3	4698.6	9340.7	4909.9	9897.5	5142.1	10,372.1	5318.6	9633.1	5017.3
O2	982	789.7	980.9	762.5	996.5	828.9	967.5	743	981.7	781
O3	0.078	0.062	0.066	0.057	0.061	0.057	0.063	0.058	0.067	0.059

^a^I1: number of doctors; I2: number of beds; I3: gross equipment expenditure in 100 million NTD;

C1: Total Surplus/deficit in 100 million NTD;

O1: combined inpatient and outpatient revenues from NHI in 100 million NTD;

O2: EBITDA value in 10 thousand

O3: (reciprocal)The rate of emergency transfer to inpatient stay over 48 h

### Dynamic efficiency of medical centers

Table 2 shows the overall dynamic efficiencies and 2015–2018 term efficiencies for each medical center, which were calculated with input-oriented models under CRS and VRS assumptions. The scale efficiency was calculated by dividing CRS by VRS. This expresses whether or not a medical center is operating at its optimal capacity. The findings indicated that 68.4% (13 of 19) of medical centers are inefficient according to CRS efficiency. Based upon the CRS assumption, the efficient medical centers active during the 2015–2018 period were hospitals B, C, D, E, G, I, and S. The DDEA model showed the mean CRS efficiencies for all hospitals are 0.85, 0.85, 0.83 and 0.87 respectively; the mean CRS efficiencies for inefficient hospitals are 0.78, 0.77, 0.76, 0.82 respectively from 2015 to 2018. The VRS scores show higher than the CRS scores and increase year by year.

**Table 2:** Efficiency Scores by Input Oriented Model

DMU	CRS						VRS						Scale					
	Overall Score	Rank	2015	2016	2017	2018	Overall Score	Rank	2015	2016	2017	2018	Overall Score	Rank	2015	2016	2017	2018
A	0.67	16	0.68	0.65	0.66	0.69	0.76	17	0.77	0.74	0.76	0.76	0.88	16	0.88	0.88	0.87	0.91
B	1.00	1	1.00	1.00	1.00	1.00	1.00	1	1.00	1.00	1.00	1.00	1.00	1	1.00	1.00	1.00	1.00
C	0.61	19	0.58	0.58	0.59	0.69	1.00	1	1.00	1.00	1.00	1.00	0.61	18	0.58	0.58	0.59	0.69
D	1.00	1	1.00	1.00	1.00	1.00	1.00	1	1.00	1.00	1.00	1.00	1.00	1	1.00	1.00	1.00	1.00
E	1.00	1	1.00	1.00	1.00	1.00	1.00	1	1.00	1.00	1.00	1.00	1.00	1	1.00	1.00	1.00	1.00
F	0.70	15	0.70	0.67	0.68	0.74	0.76	18	0.74	0.73	0.74	0.81	0.92	9	0.95	0.92	0.92	0.91
G	1.00	1	1.00	1.00	1.00	1.00	1.00	1	1.00	1.00	1.00	1.00	1.00	1	1.00	1.00	1.00	1.00
H	0.64	18	0.61	0.59	0.60	0.77	0.92	14	0.88	0.86	0.93	1.00	0.70	14	0.69	0.69	0.65	0.77
I	1.00	1	1.00	1.00	1.00	1.00	1.00	1	1.00	1.00	1.00	1.00	1.00	1	1.00	1.00	1.00	1.00
J	0.90	10	0.90	0.86	0.85	0.97	0.95	12	0.95	0.93	0.93	0.97	0.95	6	0.95	0.92	0.91	1.00
K	0.83	12	0.75	0.78	0.79	1.00	1.00	1	1.00	1.00	1.00	1.00	0.83	11	0.75	0.78	0.79	1.00
L	0.84	11	0.78	0.77	0.84	0.99	0.94	13	0.89	0.88	1.00	1.00	0.89	10	0.88	0.88	0.84	0.99
M	0.95	7	1.00	1.00	1.00	0.79	1.00	1	1.00	1.00	1.00	1.00	0.95	4	1.00	1.00	1.00	0.79
N	0.90	8	0.93	0.89	0.87	0.91	1.00	1	1.00	1.00	1.00	1.00	0.90	7	0.93	0.89	0.87	0.91
O	0.90	9	1.00	1.00	0.79	0.80	1.00	1	1.00	1.00	1.00	1.00	0.90	7	1.00	1.00	0.79	0.80
P	0.77	14	0.81	0.78	0.75	0.77	0.79	16	0.81	0.79	0.77	0.79	0.97	2	1.00	0.99	0.97	0.97
Q	0.65	17	0.64	0.66	0.62	0.66	0.72	19	0.71	0.72	0.72	0.73	0.90	5	0.90	0.92	0.86	0.90
R	0.82	13	0.70	0.82	0.78	0.82	0.85	15	0.40	0.85	0.84	0.86	0.96	2	1.75	0.96	0.93	0.95
S	1.00	1	1.00	1.00	1.00	1.00	1.00	1	1.00	1.00	1.00	1.00	1.00	1	1.00	1.00	1.00	1.00
Mean^1^	0.85		0.85	0.85	0.83	0.87	0.93		0.9	0.92	0.93	0.94	0.95		0.95	0.95	0.94	0.95
SD^1^	0.14		0.16	0.16	0.15	0.13	0.1		0.16	0.11	0.11	0.1	0.08		0.09	0.09	0.09	0.08
Mean^2^	0.78		0.78	0.77	0.76	0.82	0.83		0.77	0.81	0.84	0.87	0.92		0.96	0.92	0.89	0.93
SD^1^	0.1		0.1	0.11	0.09	0.11	0.09		0.17	0.08	0.11	0.11	0.12		0.23	0.12	0.12	0.1

^1^all hospital

^2^only inefficiency hospital

Table 3 shows the mean efficiencies by groups with respect to hospital ownership, gross medical income, and region. An ANOVA test showed significant differences between ownership in the CRS and VRS efficiency scores (F = 3.27; *p* = 0.03 and F = 6.29; *p* < 0.00). With respect to ownership, we grouped Taiwan medical centers into four hospital categories: foundation, public, university-affiliated, and religious. Some of these can be classified into two categories. In that case, medical school-affiliated hospitals were assigned priority, followed by public or foundation hospitals. The results showed that public medical centers had the lowest CRS and VRS efficiency scores, and foundation hospitals had the highest scores. However, with respect to scale efficiency, public and foundation hospitals were similar to the religious hospitals and medical school-affiliated hospitals. In terms of gross medical income classification, there was no significant difference among the medical income groups. The gross medical income (> 20 billion) category had the highest efficiency score, followed by 15–20 billion, 10–15 billion, and < 10 billion hospitals, respectively. In terms of regional classification, the mean VRS efficiency scores also differed significantly (F = 3.03; *p* = 0.05). The hospitals located in the center of Taiwan had the highest CRS and VRS efficiency scores; however, their scale efficiency was the lowest. The southern hospitals had the highest scale efficiency score but the lowest VRS efficiency.

**Table 3:** Descriptive Statistics of efficiency of input-oriented DDEA model

Group	Level	Statistics	Input Oriented			ANOVA		
			**CRS**	**VRS**	**Scale**	**F Value (*p* value) for CRS**	**F Value (*p* value) for VRS**	**F Value (*p* value) for Scale**
**Ownership**	Public	Mean	0.78	0.85	0.92	**3.27 (0.03)**	**6.29 (0.00)**	**0.03 (0.99)**
		SD	0.15	0.13	0.06			
	University-affiliated	Mean	0.85	0.94	0.93			
		SD	0.16	0.14	0.25			
	Foundation	Mean	0.92	0.99	0.92			
		SD	0.14	0.04	0.12			
	Religion hospital	Mean	0.83	0.91	0.92			
		SD	0.09	0.09	0.09			
**Gross medical Income**	> 20 billion	Mean	0.90	0.94	0.96	**1.61 (0.17)**	**2.16 (0.10)**	**1.06 (0.37)**
		SD	0.13	0.11	0.05			
	15–20 billion	Mean	0.89	0.90	0.99			
		SD	0.12	0.11	0.02			
	10–15 billion	Mean	0.87	0.90	0.98			
		SD	0.13	0.14	0.18			
	< 10 billion	Mean	0.80	0.95	0.84			
		SD	0.17	0.10	0.15			
**Region**	North	Mean	0.84	0.91	0.92	**0.39 (0.68)**	**3.03 (0.05)**	**1.36 (0.26)**
		SD	0.15	0.11	0.10			
	Center	Mean	0.88	0.99	0.89			
		SD	0.17	0.03	0.17			
	South	Mean	0.85	0.90	0.96			
		SD	0.14	0.15	0.19			

Ownership including Public Hospitals (A, D, F, Q, O); University-affiliated (B, C, G, L, R); Foundation (E, H, I, M, N, S); Religion hospital (J, K, P)

### Factors that influence hospital efficiency

Beta regression was used to determine the factors that affect the medical centers' performance by transforming the efficiency scores that lie between 0 and 1. Table 4 presents the results of the regression using the efficiency under CRS and VRS as a dependent variable. We found that under CRS model, an increase in the population in 100,000 (β = 0.033; *p* < 0.049); ratio of population aged below14 (β = 26.782; *p* < 0.024); medical surplus or deficit of appropriation in 100 million NTD (β = 0.064; *p* < 0.037); inpatient case-mix index (β = 1.688; *p* < 0.047), and bed occupancy rate (β = 8.4724; *p* < 0.000) had positive effects and increased the medical centers' efficiency. Compared to university-affiliated hospitals, foundation and religious hospitals showed significant more efficiency.

**Table 4:** The predictors of the hospital efficiency

Variable	CRS						VRS				
	Estimate	Std. Error	z value	Pr( >|z|)		Variable	Estimate	Std. Error	z value	Pr( >|z|)	
Intercept	-10.918	2.773	-3.937	0.000	***	Intercept	-19.610	2.288	-8.569	0.000	***
Pop	0.033	0.017	1.966	0.049	*	Kidp	95.825	11.625	8.243	0.000	***
Oldp	-5.941	4.934	-1.204	0.229		M_reve	0.056	0.032	1.760	0.078	
Kidp	26.782	11.899	2.251	0.024	*	Reemerg	-45.954	17.378	-2.644	0.008	**
Assets	-0.003	0.003	-0.841	0.401		NPR	0.638	0.265	2.407	0.016	*
M_reve	0.064	0.030	2.091	0.037	*	Occu	7.133	1.017	7.016	0.000	***
CMI	1.688	0.849	1.989	0.047	*	SelfPay	-0.046	0.007	-6.784	0.000	***
Occu	8.472	1.277	6.635	0.000	***	Mgain	-9.382	3.894	-2.409	0.016	*
SelfPay	-0.009	0.017	-0.524	0.601		2016	0.455	0.192	2.373	0.018	*
Public hospital	0.056	0.288	0.195	0.845		2017	0.983	0.219	4.497	0.000	***
Foundation	1.178	0.279	4.227	0.000	***	2018	1.266	0.229	5.529	0.000	***
Religious hospital	1.054	0.345	3.051	0.002	**	Public hospital	-1.125	0.250	-4.505	0.000	***
						Foundation	1.000	0.249	4.021	0.000	***
						Religious hospital	-0.232	0.254	-0.911	0.362	

^*^ < 0.05; ** < 0.001; *** < 0.000

^a^(E1)Pop: Population in 1000,000; (E2)Oldp: ratio of population aged above 65; (E3)Kip: ratio of population aged below 14; (E4)Assets: assets; (E5)M_reve: medical surplus or deficit of appropriation in 100 million NTD; (E7)Reemerg: three-day re-emergence rate; (E9)CMI: case-mix index; (E10)NPR: nurse-patient ratio; (E11)Occu: bed occupancy rate; (E12)SelfPay: patient self-pay revenues in 100 million NTD; (E13) Mgain: medical profit margin rate'

Under the VRS model, an increase in the ratio of population aged below14 (β = 95.825; *p* < 0.000); nurse-patient ratio (β = 0.638; *p* < 0.016); bed occupancy rate (β = 7.133; *p* < 0.000) had positive effects and increased the medical centers' efficiency. On the other side, rate of emergency return within 3-day (E7) (β = -45.954; *p* < 0.008); patient self-pay revenues in 100 million NTD (E12) (β = -0.046; *p* < 0.000); medical profit margin rate (β = -9.382; *p* < 0.016) had significant negative estimates and had decreased effect on the medical centers' efficiency. Based upon CRS efficiency, the results showed that efficiency in 2016, 2017 and 2018 increased significantly compared to 2015 (*p* < 0.05). Ownership also had a highly significant effect on the medical centers' efficiency. Compared to university-affiliated hospitals, public medical centers, were less efficient.

## Discussion

The efficient use of available resources is critical for medical centers to achieve high healthcare productivity under the Taiwan health system. DEA is an established method to compare hospitals' performance and provides suggestions on resource utilization [29]. In contrast, our goal in this study was to evaluate the efficiency of Taiwan medical centers using a DDEA model. This model was used to compute annual and overall efficiency scores based upon inputs, outputs, and carry-overs of all 19 medical centers from 2015 to 2018.

The efficiency examination was performed under the CRS and VRS assumptions in the input-oriented model and is shown in Table 5. We found that 68.42% (13 of 19) medical centers (A, C, F, H, J, K, L, M, N, O, P, Q, R) were inefficient compared to their peers during the study period. According to their ownership, 80% (42 of 54) of public (A, F, O and Q), 60% (3 of 5) of medical school-affiliated (C, L, and R), 50% (3 of 6) of foundation (H, M and N), and 100% (3 of 3) of religion-based medical centers (J, K, and P), were inefficient during the study period.

**Table 5:** Projections (in %) of inefficient medical centers for inputs, outputs and carry-overs

2015									2016							
	**CRS**				**VRS**				**CRS**				**VRS**			
**DMUs**	**I1**	**I2**	**I3**	**C1**	**I1**	**I2**	**I3**	**C1**	**I1**	**I2**	**I3**	**C1**	**I1**	**I2**	**I3**	**C1**
A	-7.35	-9.55	-37.77	15.56	-0.95	-9.26	-33.92	15.50	-9.67	-10.11	-42.52	5.53	-2.31	-9.81	-39.28	13.46
C	-5.26	-21.29	-54.97	0.00	0.00	0.00	0.00	0.00	-15.37	-26.60	-55.28	40.88	0.00	0.00	0.00	0.00
H	0.00	0.00	0.00	0.00	0.00	0.00	0.00	0.00	0.00	0.00	0.00	0.00	0.00	0.00	0.00	0.00
J	-0.79	-0.05	-3.51	10.38	0.00	0.00	0.00	0.00	-6.69	0.00	-1.42	0.00	0.00	0.00	0.00	0.00
K	-10.76	-10.30	-54.45	19.59	0.00	0.00	0.00	0.00	-11.52	-6.72	-47.25	0.00	0.00	0.00	0.00	0.00
L	0.00	0.00	-17.13	155.17	0.00	0.00	0.00	0.00	-10.70	0.00	-19.22	190.98	0.00	0.00	0.00	0.00
O	-33.84	-0.32	-26.85	34.33	0.00	0.00	0.00	0.00	-28.75	-0.02	-13.17	0.00	0.00	0.00	0.00	0.00
P	-8.18	0.00	-29.10	83.74	-5.83	0.00	-24.97	59.74	0.00	0.00	-8.64	143.08	0.00	0.00	-13.32	114.21
Q	-15.89	-2.65	-67.47	466.17	-0.24	-2.37	-64.39	316.66	-12.21	-3.59	-62.99	2811.37	0.00	-2.28	-58.19	1951.81
R	-16.98	-2.46	-33.10	10.46	-5.39	-0.60	-26.09	3.58	-8.36	0.00	-4.28	17.99	-1.05	0.00	0.00	16.49
Average	-9.91	-4.66	-32.44	79.54	-1.24	-1.22	-14.94	39.55	-10.33	-4.70	-25.48	320.98	-0.34	-1.21	-11.08	209.60
SD	10.38	7.04	22.15	144.21	2.32	2.92	22.03	99.14	8.17	8.48	24.06	877.65	0.77	3.11	20.76	613.17
2017									2018							
	CRS				VRS				CRS				VRS			
DMUs	I1	I2	I3	C1	I1	I2	I3	C1	I1	I2	I3	C1	I1	I2	I3	C1
A	-11.87	-6.63	-47.31	27.49	-1.05	-6.45	-41.89	25.10	-10.72	-4.36	-48.81	0.00	-0.40	-3.72	-43.39	0.87
C	-18.35	-28.05	-49.57	20.43	0.00	0.00	0.00	0.00	-20.07	-24.58	-53.92	20.54	0.00	0.00	0.00	0.00
H	-19.85	-10.81	-19.79	12.20	0.00	0.00	0.00	0.00	-6.41	-4.68	-4.88	0.00	0.00	0.00	0.00	0.00
J	0.00	0.00	0.00	0.00	0.00	0.00	0.00	0.00	0.00	0.00	0.00	0.00	0.00	0.00	0.00	0.00
K	-11.03	-1.24	-46.37	12.84	0.00	0.00	0.00	0.00	-3.62	0.00	-9.63	162.95	0.00	0.00	0.00	0.00
L	-5.01	0.00	-10.90	330.88	0.00	0.00	0.00	0.00	-3.87	0.00	-3.85	124.87	0.00	0.00	0.00	0.00
O	-31.65	0.00	-18.17	34.74	0.00	0.00	0.00	0.00	-33.33	-1.89	-21.50	26.69	0.00	0.00	0.00	0.00
P	-14.53	0.00	-28.20	100.97	-13.60	0.00	-21.81	70.51	-14.48	0.00	-18.40	204.22	-12.10	0.00	-14.91	145.30
Q	-11.42	-3.86	-62.01	371.51	0.00	0.00	-54.81	211.50	-13.34	-5.95	-61.95	188.06	-0.71	-5.37	-56.20	79.39
R	-17.60	-1.18	-22.26	21.38	-15.42	-0.94	-9.45	17.77	-20.49	-1.41	-18.26	0.00	-11.56	-0.74	-11.56	0.00
Average	-14.13	-5.18	-30.46	93.24	-3.01	-0.74	-12.80	32.49	-12.63	-4.29	-24.12	72.73	-2.48	-0.98	-12.61	22.56
SD	8.66	8.80	19.85	139.01	6.09	2.03	20.24	66.75	10.05	7.47	22.55	86.57	4.94	1.93	20.57	49.81
	**CRS**				**VRS**											
**2015–2018**	**I1**	**I2**	**I3**	**C1**	**I1**	**I2**	**I3**	**C1**								
Average	-11.75	-4.71	-28.12	141.63	-1.77	-1.04	-12.85	76.05								
SD	9.16	7.67	21.61	446.99	4.08	2.46	20.14	311.07								

^a^I1: number of doctors; I2: number of beds; I3: gross equipment expenditure in 100 million NTD

C1: medical revenue in 100 million NTD

In addition to the efficiency score, DDEA can be used to measure the proportion of the reduced inputs at a fixed level of outputs. Hence, the efficiency difference can describe the ability of a hospital to utilize resources. The DDEA projections suggested that low utilization of healthcare facilities because they were either too big or the demand was too low, reduced efficiency [30]. The projections for inefficient medical centers were obtained by input-oriented under both CRS and VRS assumptions and are shown in Table 5. During 2015–2018, the overall mean of projections under CRS indicated that making changes in such inputs as doctors (I1) by -22.59 ± 1.06%; beds (I2) by -18.95 ± 0.92%, and gross equipment expenditure in 100 million NTD (I3) by -34.56 ± 1.16% could improve the efficiency score. On the other hand, the overall mean of projections under VRS indicated that adjusting the doctors (I1) by -12.46 ± 1.07%%, beds (I2) by -17.54 ± 7.61%, and gross equipment expenditure in 100 million NTD (I3) by -25.58 ± 5.87% could increase the efficiency score. During 2015–2018, the overall mean efficiency of carry-over C1: medical revenue under CRS and VRS, was 292.48 ± 310.20% and 326.49 ± 369.78%, respectively. We found that the high projection of carry-over for hospital P in 2017 and 2018 may be due to the increased value of fixed assets and the decrease of the total surplus or deficit during that two years. Adding the carry-over connection reflected the influence of the following year's efficiency.

The results showed that the DDEA model could be a valuable tool to determine the efficiency of medical centers over time [15, 31]. This study found that six of the medical centers (6 of 19) were efficient and always remained in first position. The A, C, F, H, J, K, L, M, N, O, P, Q, and R medical centers were inefficient in CRS efficiency, and ranked 16, 19, 15, 18, 10, 12, 11, 7, 8, 9, 14, 17, and 13 respectively. Compared to 2015, most of the inefficient medical centers achieved a slight increase in scale efficiency in 2018, indicating that the change in inputs improved their efficiency (see Table 1) and also showed that all medical centers may have used their resources (labor, capital, etc.) efficiently to provide high quality healthcare services. In particular, when medical centers use their healthcare facilities optimally, they can enhance efficiency, and it is not necessary to consider their ownership.

Compared to technical efficiency under the VRS assumption (Table 2), some of the scale efficiency scores did not reach 1 and their efficiency scale needed to be adjusted. This was related to medical centers' ability to overcome the decline in the returns of scale by optimizing their structure through the wise allocation of resources, regular performance evaluations, and promotion methods under the global budget and DRG reimbursement system.

Public hospitals are constrained by more regulation from the government and the rigid organizational structure; hence it often fails to make appropriate corresponding strategies to respond to the changing environments. The association between ownership and hospital performance has been extensively examined. However, there is no consensus conclusion. Some found a significant relationship [32], while others did not [33]. Even in Taiwan, the results are also conflicting. Chen's study [34] showed that public hospitals outperformed non-public ones during the research period even though private hospitals had become larger and more group-oriented. However, Chen [35] and Yen [36] found that private hospitals have better technological efficiency than the public hospital. To make a more sophisticated analysis, we classified the ownership into the public, university-affiliated, foundation, and religious hospitals instead of only dividing it into public and private hospitals to reflect real managerial ownership in Taiwan. Our results showed that ownership have a significant effect on the efficiency of medical centers. Compared to university-affiliated hospitals, foundation and religious hospitals had higher efficiency than university-affiliated hospitals.

The relationship between health care quality and efficiency is inconsistent. Some studies showed that quality improvement increases costs and decreases technical efficiency; others suggested that quality is positively correlated with technical efficiency. Hence an improvement in the quality of care could save costs and enhance efficiency [37]. Sherman [38] mentioned that without explicitly including quality in the analysis, DEA could identify best-practice hospitals that use fewer resources even if they achieve this by providing low-quality healthcare service. In a country with NHI and under the global budget and prospective payment system, quality of care is an important issue that needs to be concerned while pursuing high operation performance. Our beta regression showed that the re-emergence rate in the same hospital within three days after hospitalization negatively affected hospital efficiency. More studies should be examined on this crucial issue in the future.

This study uses the terms efficiency, overall efficiency, and projections. Several studies have used different types of DDEA models to calculate DMUs' efficiency. For example, Li and Wang used the dynamic two-stage slacks-based measure model to determine the inefficiencies in both the productivity and profitability stages [39]. Li et al. demonstrated the virtual frontier dynamic model to calculate efficiency [40]. However, few studies in the healthcare field have adopted the DDEA model to evaluate hospital performance. The advantage of DDEA is that dynamic models feature carry-overs to describe the interdependence of consecutive periods. Therefore, one can model the carry-overs in a particular way. For example, the outputs produced in a given period can be imputed to resources used in preceding periods and the inputs consumed in a period can contribute to both current and future productions. The DDEA model refines the traditional DEA model to measure the efficiency of DMUs over time. In the future, further analysis can be conducted using the DDEA model to assess the efficiency of medical centers with different approaches.

Unlike other DEA-related studies, no previous research has used a general industry EBITDA measure (Earnings before Interest, Taxes, Depreciation and Amortization, EBITDA) in a hospital DEA analysis. EBITDA is an earnings metric that does not account for the interest, taxes, and non-cash expenses that may or may not reflect a company's ability to generate cash. Hence, when comparing two similar businesses, it is most helpful to determine a company's cash flow potential. This is the first study to incorporate EBITDA to eliminate the bias attributable to the fact that some medical centers treat significant dividends or non-operating income as pre-tax balances. It also eliminated the effect of sunk cost depreciation expense to reflect operating performance. In this study, we used public data sources; hence, the quality of the data was beyond the scope of our research. Finally, we suggest that future studies cover a linear program model to understand better the type of carry-over link used in the efficiency analysis.

## Conclusion

Under the universal coverage of Taiwan National Health Insurance, hospitals face tremendous pressures because of growing costs that affect resource allocation. Our study identified the inefficient medical centers in Taiwan and the factors that affected their efficiency. The results provide health policy administrators and hospital managers ways they can adjust their available resources to achieve high efficiency.

## Supplementary Information

**Additional file 1:** **Table S1.** Correlation between inputs and outputs for all periods of 2015-2018.

## Data Availability

The raw data are available to the following official link. https://www.nhi.gov.tw/AmountInfoWeb/TargetItem.aspx?rtype=2 https://www.ris.gov.tw/app/en/3910 https://www.mohw.gov.tw/np-129-2.htmlI The data underlying the results presented in the study are available upon request to the correspondent.
