# Supplementary material for: Evaluating the comparative efficiency of medical centers in Taiwan: a dynamic data envelopment analysis application
Source: BMC Health Serv Res. 2022 Apr 2;22:435. doi: 10.1186/s12913-022-07869-8 (PMC8976980; doi:10.1186/s12913-022-07869-8)
Supplement: Supplementary file 1 — Additional file 1: Table S1. Correlation between inputs and outputs for all periods of 2015-2018. [file 12913_2022_7869_MOESM1_ESM.docx]

**Table S1. Correlation between inputs and outputs for all periods of 2015-2018**

| **Variables**† |  | **I1** | **I2** | **I3** | **CI** | **O1** | **O2** | **O3** |
| --- | --- | --- | --- | --- | --- | --- | --- | --- |
| **I1** | Pearson | 1 | 0.940** | 0.733** | 0.976** | 0.695** | 0.711** | -0.031 |
|  | Sig. (2-tailed) |  | <.0001 | <.0001 | <.0001 | <.0001 | <.0001 | 0.7933 |
| **I2** | Pearson | 0.940** | 1 | 0.675** | 0.951** | 0.647** | 0.739** | -0.013 |
|  | Sig. (2-tailed) | <.0001 |  | <.0001 | <.0001 | <.0001 | <.0001 | 0.9117 |
| **I3** | Pearson | 0.733** | 0.675** | 1 | 0.725** | 0.372** | 0.330** | -0.077 |
|  | Sig. (2-tailed) | <.0001 | <.0001 |  | <.0001 | 0.0009 | 0.0036 | 0.5087 |
| **CI** | Pearson | 0.976** | 0.951** | 0.725** | 1 | 0.742** | 0.747** | 0.030 |
|  | Sig. (2-tailed) | <.0001 | <.0001 | <.0001 |  | <.0001 | <.0001 | 0.8 |
| **O1** | Pearson | 0.695** | 0.647** | 0.372** | 0.742** | 1 | 0.521** | 0.132 |
|  | Sig. (2-tailed) | <.0001 | <.0001 | 0.0009 | <.0001 |  | <.0001 | 0.2541 |
| **O2** | Pearson | 0.711** | 0.739** | 0.330** | 0.747** | 0.521** | 1 | 0.003 |
|  | Sig. (2-tailed) | <.0001 | <.0001 | 0.0036 | <.0001 | <.0001 |  | 0.977 |
| **O3** | Pearson | -0.031 | -0.013 | -0.077 | 0.030 | 0.132 | 0.003 | 1 |
|  | Sig. (2-tailed) | 0.793 | 0.912 | 0.509 | 0.800 | 0.254 | 0.977 |  |

† number of doctors(I1) ; number of beds (I2) ; gross equipment expenditure in 100 million NTD (I3) ; the total adjusted combined inpatient and outpatient revenues in NHI (C1) ; EBITDA index (O1) ; total revenue (O2) ; rate of emergency transfer to the inpatient stay over 48 hours (O3)
